# Interactions of major cat allergen Fel d 1 with human IgE antibody

**DOI:** 10.51894/001c.143903

**Published:** 2025-09-30

**Authors:** Kriti Khatri, Alyssa Ball, Jill Glesner, Lisa D. Vailes, Sabina Wünschmann, Scott Gabel, Geoff Mueller, Jian Zhang, R. Stokes Peebles, Martin D. Chapman, Scott A. Smith, Maksymilian Chruszcz, Anna Pomés

**Affiliations:** 1 College of Osteopathic Medicine Michigan State University

## 16

### BACKGROUND

Fel d 1 is a major allergen that is responsible for most cat allergies. Little is known about Fel d 1 interactions with human IgE (hIgE) antibodies at the molecular level. Structural studies of the Fel d 1- hIgE complexes can provide molecular level insights on in vivo interactions that trigger allergic reactions. In addition, knowledge of hIgE binding epitopes can be used to improve cat allergy therapeutics.

### OBJECTIVES

The studies aim to identify IgE epitopes on Fel d 1 by elucidating Fel d 1- hIgE complexes’ structures and design epitope-based mutants with reduced IgE binding capacity.

Methods: The hIgE mAb 15H7 was obtained using B cell hybridomas derived from allergic individuals. Recombinant IgE Fab was expressed in CHO cells. The purified 15H7 Fab was complexed with either natural or recombinant Fel d 1 and crystallized. The structural analysis of Fel d 1-15H7 Fab complex led to the design of Fel d 1 epitope-mutants which were recombinantly expressed. Purified mutants/hypoallergens were tested for their capacity to a) bind IgE from cat allergic patients using inhibition ELISA assays and b) induce passive anaphylaxis in a human FcεRI transgenic mice model study in vivo.

### RESULTS

The crystal structures of the hIgE 15H7 Fab complex with natural and recombinant Fel d 1 were determined at resolutions of 2.8 Å and 3.0 Å, respectively. Both structures revealed in vivo-like molecular interactions between heterodimeric Fel d 1 and hIgE 15H7. The epitope-mutant showed significantly reduced polyclonal IgE binding versus the wildtype in patients’ plasma and failed to induce in vivo passive anaphylaxis in mice, implying that the 15H7 mAb epitope is immunodominant and has biological significance.

### CONCLUSION

In this study, we determined the structures of Fel d 1 complexed with hIgE mAb 15H7 Fab which revealed the first structurally characterized IgE epitope of Fel d 1. The structural studies provided a rational basis for designing epitope-based Fel d 1 mutant allergens which can be used for development of hypoallergens and immunotherapy.

